# Analysis of Pharmacogenomic Variants Associated with Population Differentiation

**DOI:** 10.1371/journal.pone.0119994

**Published:** 2015-03-25

**Authors:** Bora Yeon, Eunyong Ahn, Kyung-Im Kim, In-Wha Kim, Jung Mi Oh, Taesung Park

**Affiliations:** 1 Interdisciplinary Program in Bioinformatics, Seoul National University, Gwanak-ro, Gwanak-gu, Seoul, Korea; 2 College of Pharmacy, Seoul National University, Gwanak-ro, Gwanak-gu, Seoul, Korea; 3 Department of Statistics, Seoul National University, Seoul, Korea; University of Bristol, UNITED KINGDOM

## Abstract

In the present study, we systematically investigated population differentiation of drug-related (DR) genes in order to identify common genetic features underlying population-specific responses to drugs. To do so, we used the International HapMap project release 27 Data and Pharmacogenomics Knowledge Base (PharmGKB) database. First, we compared four measures for assessing population differentiation: the chi-square test, the analysis of variance (ANOVA) F-test, F_st_, and Nearest Shrunken Centroid Method (NSCM). F_st_ showed high sensitivity with stable specificity among varying sample sizes; thus, we selected F_st_ for determining population differentiation. Second, we divided DR genes from PharmGKB into two groups based on the degree of population differentiation as assessed by F_st_: genes with a high level of differentiation (HD gene group) and genes with a low level of differentiation (LD gene group). Last, we conducted a gene ontology (GO) analysis and pathway analysis. Using all genes in the human genome as the background, the GO analysis and pathway analysis of the HD genes identified terms related to cell communication. “Cell communication” and “cell-cell signaling” had the lowest Benjamini-Hochberg’s q-values (0.0002 and 0.0006, respectively), and “drug binding” was highly enriched (16.51) despite its relatively high q-value (0.0142). Among the 17 genes related to cell communication identified in the HD gene group, five genes (*STX4*, *PPARD*, *DCK*, *GRIK4*, and *DRD3*) contained single nucleotide polymorphisms with F_st_ values greater than 0.5. Specifically, the F_st_ values for rs10871454, rs6922548, rs3775289, rs1954787, and rs167771 were 0.682, 0.620, 0.573, 0.531, and 0.510, respectively. In the analysis using DR genes as the background, the HD gene group contained six significant terms. Five were related to reproduction, and one was “Wnt signaling pathway,” which has been implicated in cancer. Our analysis suggests that the HD gene group from PharmGKB is associated with cell communication and drug binding.

## Introduction

The DNA sequence of the 3-billion-nucleotide-long human genome varies by approximately 0.1% between individuals. Surprisingly, this small difference in the DNA sequence accounts for individual differences in appearance, behavior, and even disease status. Furthermore, this difference in DNA sequence can have an even larger effect among ethnic populations. Genetic divergence between ethnic groups is called population differentiation (PD). PD results from genetic factors that enforce natural selection, genetic drift, or gene flow. Moreover, genes related to Mendelian diseases have a significant excess of single-nucleotide polymorphisms (SNPs) with high levels of PD, and the incidence of and susceptibility to these diseases differ among populations [1].

Several recent studies on PD have focused on genetic variations. Myles *et al*. attempted to identify SNPs accounting for disease-associated PD [2]. However, they found no disease-associated SNPs that were more significantly differentiated than randomly selected SNPs in the genome among populations. Nevertheless, the frequencies of risk alleles for disease-associated SNPs showed substantial variation across human populations. Barreiro *et al*. analyzed the degree of PD with 2.8 million SNPs and discovered the role of natural selection in shaping PD [3]. Wu and Zhang also performed a genome-wide study of PD and found that many groups of genes had higher degrees of PD [1]. Specifically, PD existed on some loci associated with phenotypes (e.g., hair growth and pigmentation) that are well known to vary across populations.

PD has also been investigated in pharmacogenomic studies [4]. For example, the response to warfarin, one of the most widely studied drugs, depends not only on genetic variants [5] but also on population [6]. As a result, some authors have suggested that warfarin be dosed according to the patient’s race. In fact, Pavani *et al*. suggested a linear model for optimizing population-specific warfarin dose [7]. Huang *et al*. identified SNPs contributing to etoposide-induced cytotoxicity in a genome-wide association study (GWAS) using International HapMap cell lines, and they demonstrated different genotypes associated with cytotoxicity between two populations [8]. In order to investigate PD of DR genes, we analyzed data from two databases: International HapMap release 27 (phase II + III) [9] and Pharmacogenomics Knowledge Base (PharmGKB) [10,11], the most widely used DR database. Originally, HapMap release 27 contained 11 subpopulations. However, the allele frequencies of populations in the same ethnic groups are highly correlated [12], and there is lack of genotypic information in some populations. Therefore, we used the following subpopulations: European, African, and Asian from Japan and China.

There are several measures for determining the distance among populations. Among them, F_st_ is the most widely used measure to determine PD. Akey *et al*. [13] and Barreiro *et al*. [3] used Weir and Cockerham’s estimate, an unbiased estimate of F_st_ [14,15]. Casto *et al*. used four measures: (i) δ, the difference in allele frequency between two groups; (ii) integrated haplotype score (iHS), which characterizes the lengths of the haplotypes surrounding each allele of a SNP [16]; (iii) latitude/longitude correlation (LLC), which describes how closely changes in a SNP’s allele frequency follow geographical coordinates; and (iv) F_st_, which shows variation in allele frequency among populations [17]. Park *et al*. used the Nearest Shrunken Centroid Method (NSCM) [18,19], which was originally designed for clustering of microarray data. NSCM has been proposed for solving the classification problem with a large number of features and it was also applied to the analysis of population differentiation in SNPs via Hapmap data [18]. Han *et al*. modified F_st_ for use with allele frequency data with unbalanced sample sizes [20].

In order to investigate PD of DR genes, we first compared four measures for assessing population differentiation: the chi-square test, the ANOVA F-test, F_st_, and NSCM. F_st_ showed high sensitivity with stable specificity among varying sample sizes; thus, we selected F_st_ for determining population differentiation. We then divided DR genes from PharmGKB into two groups based on the degree of population differentiation as assessed by F_st_: genes with high a level of differentiation (HD gene group) and genes with a low level of differentiation (LD gene group). Finally, we conducted a gene ontology (GO) analysis and pathway analysis.

Several studies have investigated PD associated with individual drugs [4]. In the present study, we systematically studied PD of drug-related (DR) genes by simultaneously considering all reported DR genes. This integrative approach may help clarify the inconsistent genetic features of drug response associated with PD. Furthermore, our findings will improve the study and prediction of drug responses that differ among populations due to genetic stratification.

## Methods

### 1. Measures of PD

Since the measures of PD are not always consistent, it is difficult to choose an appropriate measure for PD. Thus, we first performed a comparison analysis in order to identify the highest performing measures in our study. We compared the following four measures: Weir and Cockerham’s F_st_ [15], the sum of square of *d*_*i*_ from NSCM, the chi-square test, and analysis of variance (ANOVA) F-test. Other measures were excluded for the following reasons. δ is used for comparisons between two populations; however, we compared PD among three populations. In our research, we tried to evaluate which SNPs are highly differentiated but iHS shows whether SNPs are differently selected. Therefore, the results via iHS are not concordant with the results from other measures. Moreover, Ferrer-Admetlla et al. suggest that iHS seems to be affected by the recombination rate [21]. Thus, we would like to exclude iHS from our sensitivity and specificity analysis. LLC was excluded, because latitude and longitude information for each individual was needed to determine PD.

We compared the specificity and sensitivity of these measures using simulation studies. Our comparison study focused on consistency and reliability with respect to the populations’ sample sizes and imbalance in sample sizes among populations. Our comparison revealed that F_st_ had the most stable specificity regardless of the variability in sample size and the highest sensitivity as compared to other measures. Thus, we concluded that F_st_ was the most appropriate measure of PD for our integrative analysis of International HapMap release 27 and PharmGKB.

The chi-square test is a widely used statistical method for testing the homogeneity of group proportions. In this study, we used it to test whether allele frequencies of the *J* subgroups were equal; the null hypothesis was:

$$p_{1}=\cdots =p_{J}$$ (1)

where *p*_*i*_ denotes the allele frequency of the *i*_th_ population. In the chi-square test, 0.05 or the q-value from Benjamini and Hochberg’s method [22] is usually used as the significance level for testing the null hypothesis. Thus, the significance level varies according to *N*.

The model for the ANOVA F-test was:

$$a_{ij}=\mu +\tau_{i}+{\overset{´}{o}}_{ij},\overset{}{\sum }\tau_{i}=0$$ (2)

where *a*_*ij*_ is the number of the allele (value of 0, 1, or 2) for the *j*_th_ individual in the *i*_th_ population. *μ* and *μ*+*τ*_*i*_ are the overall mean genotype frequencies within individuals and mean of allele frequencies in the *i*_th_ population, respectively. *ε*_*ij*_ is the error term. Thus, by testing H_0_:*τ*_*i*_ = 0, for ^∀^ *i*, we could test whether the allele frequencies of subgroups were equal to one another assuming a Gaussian distribution.

We used Weir’s F_st_ estimate$\overset{\wedge }{\theta }$ [14,15], an unbiased estimator of F_st_. *n*_*i*_ denotes the sample size of the *i*_th_ subpopulation (i = 1,…,*s*). n = Σ*n*_*i*_ denotes the total sample size. $\hat{p_{i}}$denotes the observed allele frequency of the *i*_th_ subpopulation, and $\bar{p}=\sum^{}n_{i}\hat{p_{i}}/n$ denotes the weighted average of allele frequency.

$$\hat{\theta }=\frac{\text{MSP}-\text{MSG}}{\text{MSP}+\left({\text{n}}_{\text{c}}-1\right)\text{MSG}},$$ (3)

where

$$\text{MSP}=\frac{1}{s-1}\overset{s}{\underset{i=1}{\sum }}n_{i}{\left(\hat{p_{i}}-\bar{p}\right)}^{2},$$ (4)

$$\text{MSG}=\frac{1}{\sum_{i=1}^{s}(n_{i}-1)}\overset{s}{\underset{i=1}{\sum }}n_{i}\hat{p_{i}}\left(1-\hat{p_{i}}\right),$$ (5)

$$n_{c}=\frac{1}{s-1}\left(\overset{s}{\underset{i=1}{\sum }}n_{i}-\frac{\sum_{i=1}^{s}n_{i}^{2}}{\sum_{i=1}^{s}n_{i}}\right)$$ (6)

MSP and MSG represent the observed mean square error of a locus between populations and the observed mean square error of a locus within populations, respectively. *n*_*c*_ is the average sample size across *n* samples, correcting for variation in sample size among subpopulations.

We also defined the sum of square of standardized distance to measure PD via NSCM as follows;

$${\text{SS}}_{\text{d}}=\underset{i}{\sum }d_{ik}^{2}$$ (7)

It is a representative value for the *k*_*th*_ SNP in population *i*, where

$$d_{ik}=\frac{a_{ik}-a_{k}}{m_{i}\left(s_{0}+s_{k}\right)}$$ (8)

Here, *a*_*ik*_ denotes the mean of allele frequencies in population *i*; *a*_*k*_ denotes the overall mean of allele frequency of SNP *k*, and $m_{i}=\sqrt{\frac{1}{n_{k}}+\frac{1}{n}}$, which makes *m*_*i*_·*s*_*k*_ equal to the estimate of standard error for the numerator of *d*_*ik*_·*s*_0_ was set equal to the median of *s*_*k*_ over the set of SNPs to prevent inflation of *d*_*ik*_.

### 2. GO analysis and pathway analysis of the HD and LD gene groups

We used a gene ontology (GO) analysis to identify biological characteristics of the HD and LD gene groups. We compared each gene group to other functionally annotated genes in HapMap Data [23] and to DR genes in the PharmGKB Database.

Wright proposed the following F_st_ categories: (i) F_st_ < 0.05, low divergence; (ii) 0.05 < F_st_ < 0.15, moderate divergence; (iii) 0.15 < F_st_ < 0.25, high divergence; and (iv) F_st_ > 0.25, very high divergence [24]. Using Wright’s F_st_ criteria, genes containing at least one SNP with an F_st_ value greater than 0.25 were considered to have a high level of differentiation (HD gene group) [25,26], while those containing SNPs with F_st_ values less than 0.05 were considered to have a low level of differentiation (LD gene group). Additionally, we identified the SNPs with a high level of differentiation from GO analysis results if F_st_ greater than 0.5, because this criterion was used for previous studies [1,27].

For the GO analysis, SNPs associated with drugs from PharmGKB were annotated into genes. These DR genes were divided into two groups using the F_st_ criteria proposed by Wright [24]. From 654 DR SNPs in the HapMap Database, we obtained 160 SNPs with HD and 173 SNPs with LD (Table 1). From these SNPs, 68 genes with HD and 114 genes with LD were derived.

**Table 1:** Summary of each SNP group.

	SNPs with high differentiation	SNPs with low differentiation	Total
**Count**	160	173	654
**Mean (Fst)**	0.364	0.020	0.157
**Median (Fst)**	0.344	0.019	0.111

To investigate the biological differences between the HD and LD gene groups, we performed a GO analysis and a pathway analysis using the Database for Annotation, Visualization and Integrated Discovery (DAVID) [28] v6.7 functional annotation tool. Annotated genes from each group were used as the input, while a list of whole genes in DAVID with at least one annotation in the analyzing categories was used as the background. For the GO analysis, the following three categories were selected: biological process (BP), molecular function (MF), and cellular component (CC) [29]. For the pathway analysis, the Kyoto Encyclopedia of Genes and Genomes (KEGG) pathway was used [30].

Additional GO and pathway analyses were performed in a similar manner in order to compare genes in the HD gene group to those in the DR gene group. In this case, the DR HD gene group was used as the input for analysis, and the DR gene group was used as the background.

To correct for multiple tests, we used the hypergeometric test from Benjamini-Hochberg’s method [22]. Fold enrichments, defined as the ratios of proportions between the input and background, were calculated for each term. Terms with Benjamini-Hochberg’s q-values of 0.05 or lower were considered significant.

## Results

### 1. Data collection

**Fig 1:**
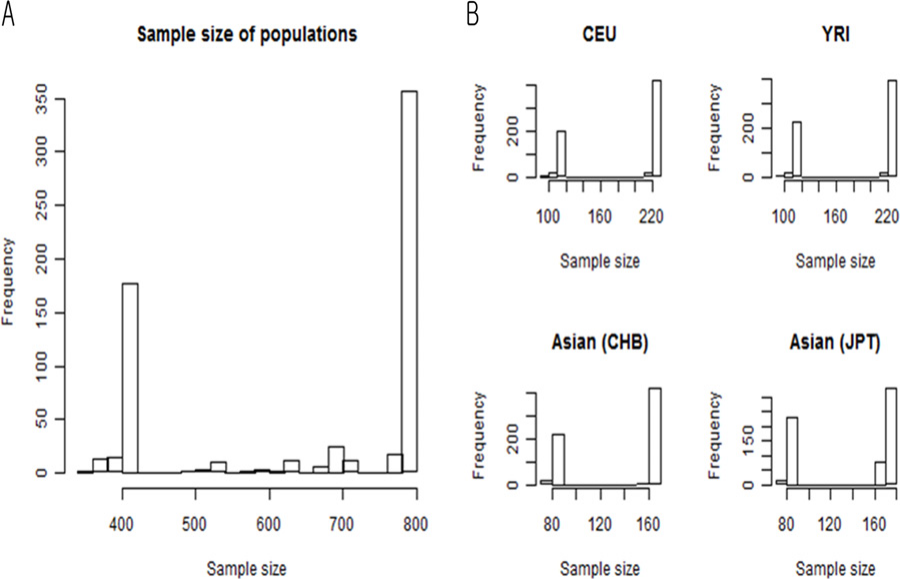
Histogram of sample sizes from 654 drug-related SNPs. **A**. Total sample sizes of SNPs. **B**. Sample size of each population of SNPs. CHB and JPT are plotted separately according to the format of the original HapMap Data. SNPs with larger sample sizes are included in Phase III, and SNPs with smaller sample sizes are included in Phase II.

We analyzed SNP data from International HapMap Phase II + III, release 27 (http://www.hapmap.org) [9,31]. According to Hapmap consortium, there are distinct three clusters, European, African and Asian from the principal component plot of 11 populations in hapmap3[31]. Therefore, we used three groups based on these three regions. We included 120 Yoruba from Ibadan, Nigeria (YRI), 181 Asians of which are 91 Japanese from Tokyo, Japan (JPT) and 90 Han Chinese from Beijing, China (CHB), and 120 Utah residents with ancestry from northern and western Europe (CEU). We used only founders of CEU and YRI to exclude the related samples. Because International HapMap release 27 consists of a mixture of two phases, each SNP had a different sample size. Fig. 1 shows the sample-size distributions of subpopulations from International HapMap Data. The SNPs from Phase II had smaller sample sizes, while those from Phase III had larger sample sizes (Fig. 1B). Some SNPs are only genotyped in phase II and others are only genotyped in phase III. In this reason, we only included four populations, which are both in phase II, and III simultaneously to avoid the potential biases due to different settings of each phases.

In addition, Kim et al. (2012) reported that JPT and CHB in Hapmap possess the common genetic information through MDS plot and Fst [32]. Therefore, we merged these two data into one East Asian data. We used allele frequency data of three populations as following: Yoruba in Ibadan, Nigeria (YRI), Utah residents with ancestry from Northern and Western Europe (CEU), and East Asian (EA), which consists of the Han Chinese population in Beijing, China (CHB) and the Japanese population in Tokyo, Japan (JPT). Also, we used only founders of CEU and YRI to exclude the related samples. For pharmacological research, we collected 2595 DR SNPs from PharmGKB (http://www.pharmgkb.org) [10,11]. Finally, from these two databases, 654 compatible SNPs among three populations were used for analysis.

### 2. Comparison of four PD measures using simulation

We selected four PD measures: chi-square test, Weir’s F_st_, ANOVA F-test, and sum of square of *d*_*i*_ from NSCM. Since each phase in HapMap release 27 had different sample sizes, we set the sample size *n*_*i*_ of the subpopulations as a parameter of the simulation as well as the distance *d* between allele frequencies in order to compare the performance of the four measures. To examine the effect of sample size on these measures, we set *n*_*i*_ as follows:

Scenario I: Increased sample sizes (*n*_1_,*n*_2_,*n*_3_) = (100,100,100), (200,200,200), and (400,400,400).

Scenario II: Unbalanced sample sizes among the subpopulations (*n*_1_,*n*_2_,*n*_3_) = (200,100,100),(100,200,100), and (100,100,200)

For convenience, we assumed equal distance between adjacent alleles and let the distance *d* = *p*_2_-*p*_1_ = *p*_3_-*p*_2_(*p*_1_≤*p*_2_≤*p*_3_). We generated *p*_*i*_ with *d* = 0.1,0.2,0.3 and *p*_1_ was generated under uniform distribution on [0, min(0.5,1-2d)] (Table 2). Here, we used *min*(0.5,1-2*d*) as the maximum of *p*_1_ rather than 1 because of the symmetry in allele frequency. If *p*_1_ is greater than 0.5, then *p*_2_ and *p*_3_ are also greater than 0.5, and we can alternatively identify a set of allele frequencies {1-*p*_3_,1-*p*_2_, *and* 1-*p*_1_} instead of {*p*_1_, *p*_2_, *p*_3_}.

**Table 2:** Examples of data sets.

*p*_1_	d		
	0.1	0.2	0.3
0	{0, 0.1, 0.2}	{0, 0.2, 0.4}	{0, 0.3, 0.6}
0.1	{0.1, 0.2, 0.3}	{0.1, 0.3, 0.5}	{0.1, 0.4, 0.7}
0.2	{0.2, 0.3, 0.4}	{0.2, 0.4, 0.6}	{0.2, 0.5, 0.8}
0.3	{0.3, 0.4, 0.5}	{0.3, 0.5, 0.7}	{0.3, 0.6, 0.9}
0.4	{0.4, 0.5, 0.6}	{0.4, 0.6, 0.8}	{0.4, 0.7, 1.0}
0.5	{0.5, 0.6, 0.7}	{0.5, 0.7, 0.9}	NA

**Fig 2:**
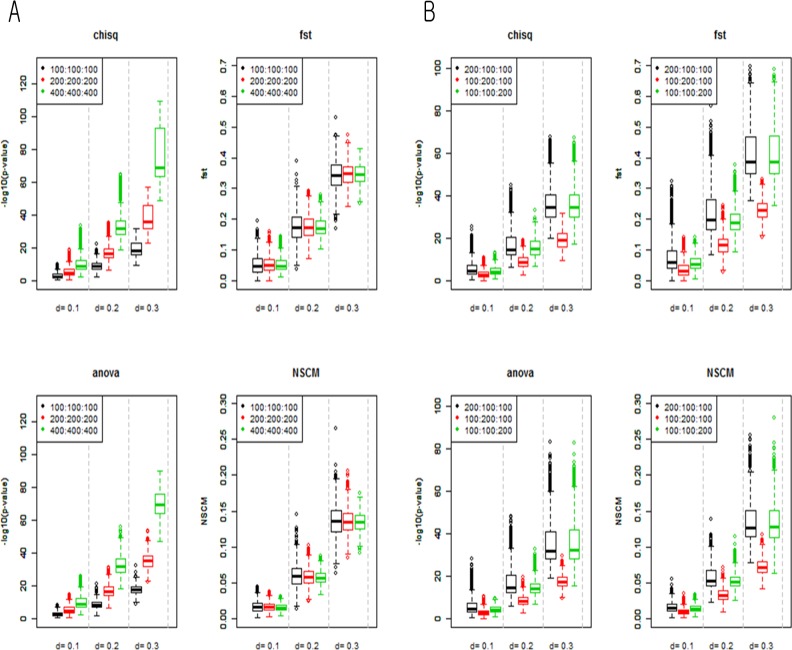
Boxplots representing four measures of simulation data with an increase in *d*. **A.** Variation of distributions due to increase in sample sizes (Case I). **B.** Variation of distributions due to bias of sample sizes (Case II). For both cases, the *x*-axis denotes the distance *d*, and the *y*-axis and denotes the following measures: -log_10_ *Pvalue* for chi-square test and ANOVA F-test; Weir and Cockerham’s F_st_ estimates for F_st_; $\sum^{}d_{i}^{2}$ for NSCM.

From these conditions, we generated 200 sets of genotype frequency data from three binomial distributions under Hardy-Weinberg Equilibrium (HWE): binomial distribution$\left(n_{i},p_{i}^{2}\right)$, binomial distribution (*n*_*i*_, 2*p*_*i*_ *q*_*i*_) and binomial distribution$\left(n_{i},q_{i}^{2}\right)$. We then calculated the four PD measures. Fig. 2 shows the box plots representing the distributions of the measures from two scenarios. As *d* increased, the box sizes of the chi-square test, ANOVA F-test, and NSCM increased, while those of F_st_ did not. All measures increased as *d* increased. As the total sample size increased, the *p*-values from the chi-square test and ANOVA F-test decreased, while those from F_st_ and SS_d_ from NSCM did not change significantly (Fig. 2A).

Fig. 2B shows the effect of unbalanced sample size. All measures tended to increase as *d* increased. All measures showed higher levels of differentiation when *n*_1_ or *n*_3_ was unbalanced (*n*_1_,*n*_2_,*n*_3_) = (200,100,100)·(100,100,200) than when *n*_2_ was unbalanced (*n*_1_,*n*_2_,*n*_3_) = (100,200,100). This was because sample sizes *n*_1_ and *n*_3_ of the subpopulation with extreme values of allele frequencies *p*_1_ and *p*_3_ had large effects on the PD measures.

### 3. Comparison of the sensitivity and specificity of four PD measures

We simulated additional data from the same sample-size condition *n*_*i*_ as described above. *p*_1_ was generated under uniform distribution on [0, min(0.5,1-2d)]. In these simulations, *d* = 0 indicates the null hypothesis, and other values of *d* indicate the alternative hypothesis. For a given *d* and *n_i_*, we generated 100 datasets from the three binomial distributions under HWE. We calculated the specificity (when *d* = 0) and the sensitivity (when *d* = 0.05,0.1,…,0.3) by counting the true negatives and true positives and repeated this step 100 times to calculate the average sensitivity and specificity.

**Fig 3:**
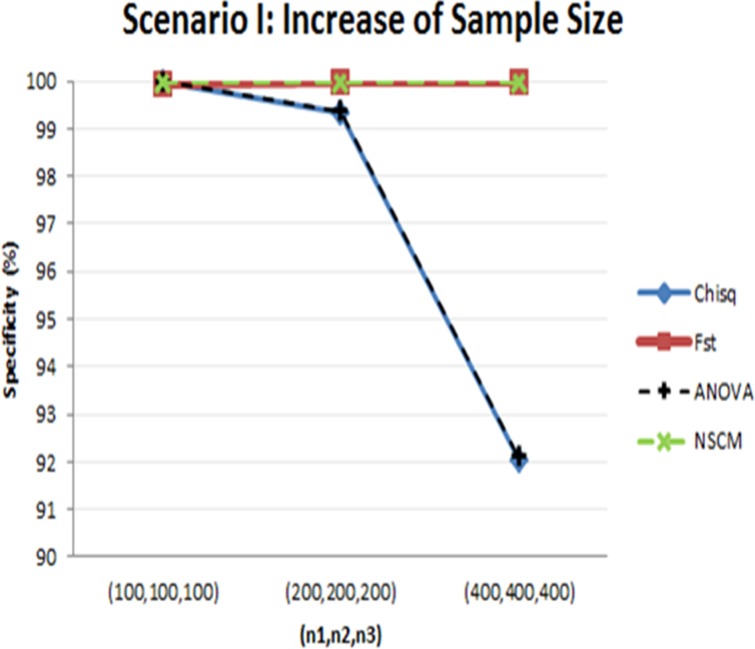
Specificities (%) of each measure from simulation data under H_0_:*d* = 0 due to an increase in sample size (Scenario I). The chi-square test and ANOVA F-test are similar, and F_st_ and SS_d_ from NSCM are nearly identical. Blue line: chi-square test; red line: F_st_; black dotted line: ANOVA F-test; green dotted line: SS_d_ from NSCM.

The chi-square test and ANOVA F-test depended on total sample sizes, as indicated by the specificities calculated under the null hypothesis (*d* = 0) for Scenario I (Fig. 3). When the total sample sizes were small, the chi-square test and ANOVA F-test showed high specificities; however, the specificities fell to 92% as the sample size increased. This reflects a general characteristic of test statistics, where the test statistic tends to reject the null hypothesis more when the sample size increases. For Scenario II, all four measures yielded high specificities that were close to one.

**Fig 4:**
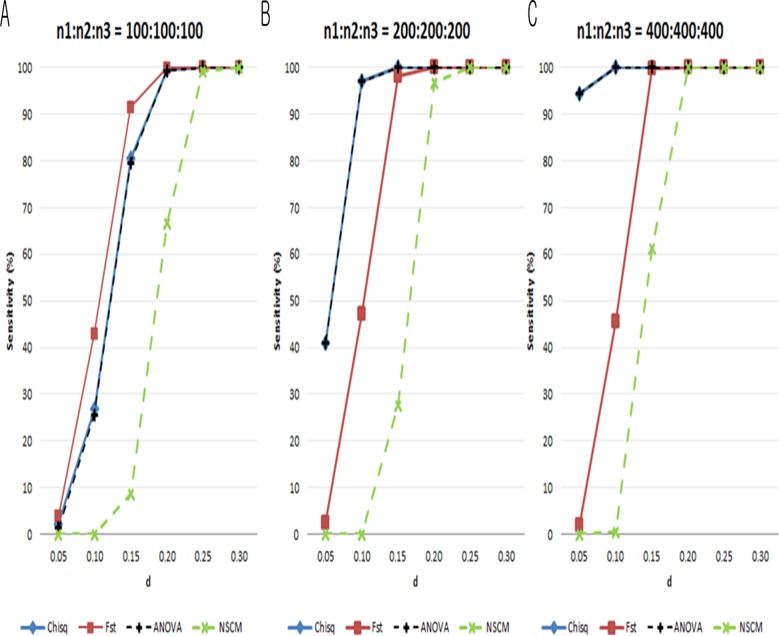
Sensitivities (%) of each measure from simulation data under H_0_:*d* = 0.05,0.1…,0.3 due to an increase in sample size (Scenario I). **A**. (*n*_1_,*n*_2_,*n*_3_) = (100,100,100). **B**. (*n*_1_,*n*_2_,*n*_3_) = (200,200,200). **C**. (*n*_1_,*n*_2_,*n*_3_) = (100,100,100). Blue line: chi-square test; red line: F_st_; black dotted line: ANOVA F-test; green dotted line: SS_d_ from NSCM.

**Fig 5:**
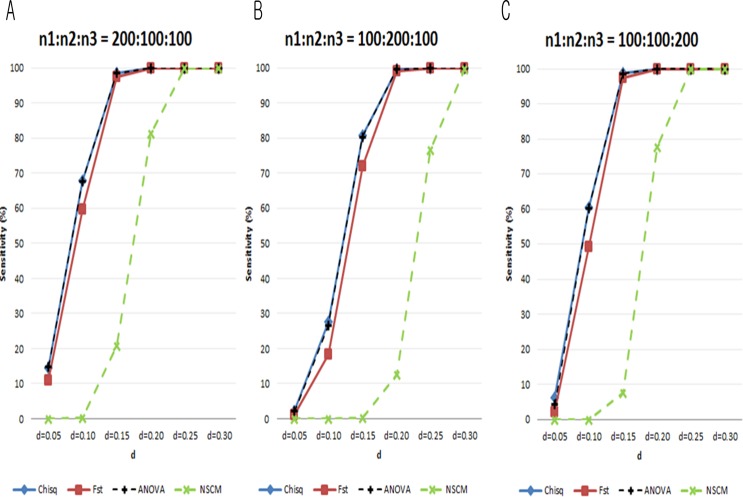
Sensitivities (%) of each measure from simulation data under H_0_:*d*=0.05,0.1…,0.3 due to bias in sample size (Scenario II). A. (*n*_1_,*n*_2_,*n*_3_) = (200,100,100). **B**. (*n*_1_,*n*_2_,*n*_3_) = (100,200,100). **C**. (*n*_1_,*n*_2_,*n*_3_) = (100,100,200). Blue line: chi-square test; red line: F_st_; black dotted line: ANOVA F-test; green dotted line: SS_d_ from NSCM.

In general, the sensitivity increased for all measures as the sample size increased. Still, NSCM consistently yielded the lowest sensitivity. The sensitivities of the chi-square test and ANOVA F-test increased as the total sample size increased. When the sample size was small, F_st_ had the highest sensitivity among the measures (Fig. 4A). When the sample size was moderate or large, the chi-square test and the ANOVA F-test had the highest sensitivities (Fig. 4B, 4C). Note that F_st_ yielded sensitivity and specificity that were robust to sample size, while the other measures did not. Fig. 5 shows the sensitivities from Scenario II. For the same *d*, specificities were lower when (*n*_1_,*n*_2_,*n*_3_) = (100,200,100) than other situations, similar to the result shown in Fig. 2B. NCSM had the lowest sensitivity. The chi-square test and ANOVA F-test had approximately equal sensitivities, while F_st_ had slightly lower sensitivities.

Based on the simulation results, we chose F_st_ for our study for the following reasons. First, the chi-square test and the ANOVA F-test were not appropriate for our data because of their dependence on sample size (Fig. 2A) and low specificity (Fig. 3); this would result in rejecting SNPs without population differences at large sample sizes. NCSM yielded the lowest sensitivity (Fig. 4). Therefore, we chose F_st_ for its high specificity and sensitivity robust to sample size.

### 4. GO analysis and pathway analysis: comparison of HD and LD gene groups with all genes in DAVID

In order to biologically interpret the HD and LD gene groups, we performed a GO analysis and pathway analysis. Eighteen terms were statistically significant when the HD gene group was analyzed independently, and 48 terms were significant when the LD gene group was analyzed independently. The separate analyses had 25 significant terms in common. Table 3 shows Benjamini-Hochberg’s q-values [22] and fold enrichments for GO terms and pathways that were statistically significant in the analysis of the HD gene group only. Table 4 shows the results for the LD gene group. When the LD gene group was used as input for the analysis (Table 4), DR terms in GO categories “drug metabolic process,” “drug metabolism,” and “metabolism of xenobiotics by cytochrome P450” were significant. The term “drug binding” was the only significant DR term resulting from analysis of the HD gene group (Table 3). From analysis of the HD gene group, several terms associated with cell communication had significant *p*-values (0.0006, 0.0002, and 0.0142) and high fold enrichments. In particular, the terms, “cell communication” and “cell-cell signaling” had the lowest *p*-values among the terms in Table 3. However, in the LD gene group, only one significant term was related to cell communication (“regulation of cell communication”), which had a q-value (0.0273) and fold enrichment (2.41).

**Table 3:** Q-values and fold enrichments of significant terms in HD group.

Terms	FE	BH’s q	Terms	FE	BH’s q
BP: Behavioral response to nicotine	96.16	0.0201	BP: Secretion	5.24	0.0466
MF: Drug binding	16.51	0.0142	BP: Regulation of cell differentiation	4.56	0.0157
BP: Adult behavior	15.65	0.0075	BP: Cell Communication	4.53	0.0002
BP: Regulation of multicellular organism growth	14.71	0.0465	BP: Cell development	3.48	0.0464
CC: Dendrite	9.63	0.0027	BP: Regulation of developmental process	3.34	0.0276
BP: Sex differentiation	8.92	0.0210	BP: Neurological system process	3.15	0.0060
BP: Development of primary sexual characteristics	8.83	0.0468	BP: Anatomical structure morphogenesis	2.81	0.0197
BP: Reproductive developmental process	5.99	0.0307	BP: Cell differentiation	2.47	0.0214
BP: Cell-cell signaling	5.24	0.0006	BP: System development	2.21	0.0136

FE: Fold enrichment

BH’s q: Benjamini-Hochberg’s q-value

**Table 4:** Q-values and fold enrichments of significant terms in LD group.

Terms	FE	BH’s q	Terms	FE	BH’s q
BP: Negative Regulation Of Amine Transport	58.70	0.0170	BP: Regulation Of Response To Stress	5.71	0.0017
*BP: Drug Metabolic Process	48.92	0.0003	BP: Positive Regulation Of Transport	5.62	0.0103
BP: Negative Regulation Of Glucose Transport	46.96	0.0253	CC: Membrane fraction	5.33	4.4E-12
BP: Negative Regulation Of Organic Acid Transport	46.96	0.0253	CC: Insoluble fraction	5.31	3.0E-12
BP: Multicellular Organismal Water Homeostasis	42.69	0.0281	BP: Heterocycle Metabolic Process	5.16	0.0017
BP: Body Fluid Secretion	28.99	0.0011	BP: Positive Regulation Of Multicellular Organismal Process	5.13	0.0144
BP: Negative Regulation Of Ion Transport	27.23	0.0082	BP: Regulation Of Anatomical Structure Morphogenesis	5.00	0.0329
MF: Oxygen Binding	26.82	0.0000	BP: Cellular Chemical Homeostasis	4.94	0.0011
BP: Renal System Process	22.36	0.0126	CC: Cell projection part	4.91	0.0173
CC: Presynaptic membrane	19.81	0.0167	CC: Microsome	4.85	0.0173
BP: Regulation Of Tube Size	17.72	0.0012	CC: Vesicular fraction	4.71	0.0190
KEGG: Linoleic acid metabolism	14.24	0.0306	KEGG: Calcium signaling pathway	4.53	0.0217
*KEGG: Drug metabolism: other enzymes	13.91	0.0024	CC: Cell fraction	4.51	3.1E-12
KEGG: Arachidonic acid metabolism	12.46	0.0013	BP: Anatomical Structure Formation Involved In Morphogenesis	4.00	0.0257
MF: Tetrapyrrole Binding	11.49	0.0000	BP: Transmembrane Transport	3.85	0.0018
KEGG: Retinol metabolism	11.08	0.0048	MF: Oxidoreductase Activity	3.83	0.0002
*KEGG: Metabolism of xenobiotics by cytochrome P450	9.97	0.0060	BP: Regulation Of Response To Stimulus	3.70	0.0124
*KEGG: Drug metabolism: cytochrome p450	9.65	0.0056	BP: Ion Transport	3.67	0.0005
BP: Negative Regulation Of Multicellular Organismal Process	8.59	0.0006	BP: Positive Regulation Of Molecular Function	3.47	0.0065
BP: Negative Regulation Of Transport	8.12	0.0051	BP: Homeostatic Process	3.13	0.0055
BP: Regulation Of Response To External Stimulus	7.88	0.0019	BP: Regulation Of Multicellular Organismal Process	3.01	0.0018
BP: Regulation Of Body Fluid Levels	6.66	0.0281	BP: Regulation Of Catalytic Activity	2.78	0.0133
BP: Muscle System Process	6.52	0.0128	CC: Endomembrane system	2.75	0.0169
BP: Angiogenesis	6.35	0.0317	BP: Regulation Of Cell Communication	2.41	0.0273

FE: Fold enrichment

BH’s q: Benjamini-Hochberg’s q-value

Seventeen genes were associated with the three terms related to cell communication (“cell-cell signaling,” “cell communication,” and “drug binding”). Among these 17 genes, we extracted five that contained SNPs with F_st_ values greater than 0.5: *STX4*, *PPARD*, *DCK*, *GRIK4*, *and DRD3* contained SNPs rs10871454, rs6922548, rs3775289, rs1954787, and rs167771 with F_st_ values of 0.682, 0.620, 0.573, 0.531, and 0.510, respectively.

Syntaxin 4 (STX4) is a component of the SNARE complex, which mediates docking of cellular transport vesicles. In a GWAS, rs10871454 in *STX4* accounted for over 25% of the variance in log-transformed stabilized warfarin dose and was in perfect linkage disequilibrium with rs9923231 [33].

PPARs are nuclear hormone receptors that bind peroxisome proliferators and control the size and number of peroxisomes produced by cells. In particular, PPARδ is a receptor that binds peroxisome proliferators such as hypo-lipidemic drugs and fatty acids [34]. The SNP rs6922548 in *PPARδ* is also associated with positive clinical response to docetaxel and thalidomide [35].

DCK is involved in the gemcitabine and lamivudine pathways [36,37]. It also participates in the phosphorylation of cytosolic nucleosides by deoxycytidine kinase and pyrimidine salvage reactions. Fukunaga *et al*. examined variants that were identified from a screen of 13 genes in the gemcitabine metabolic pathway [38] and found that the C allele of SNP rs3775289 was not present among Europeans or Africans in their study. However, in the International HapMap Data, the allelic frequencies of the C allele are 0.929 in Europeans and 0.279 in Africans.

Paddock *et al*. implemented an association study based on the Sequenced Treatment Alternatives to Relieve Depression (STAR*D) cohort and found that rs1954787 in the *GRIK4* gene, which encodes the kainic acid-type glutamate receptor KA1, was associated with response to the antidepressant citalopram [39]. Accordingly, they suggested that the glutamate system plays an important role in modulating response to selective serotonin reuptake inhibitors (SSRIs). In addition, Pickard *et al*. showed that variation in *GRIK4* was significantly associated with both an increased risk of schizophrenia and a decreased risk of bipolar disorder [40]. Furthermore, the G variant of SNP rs167771 in *DRD3* was associated with an increased risk of extrapyramidal symptoms (EPS) in psychiatric patients receiving risperidone [41].

Several terms related to reproduction were identified in the analysis of the HD gene group, but none were identified in the analysis of the LD gene group. For example, Table 3 reports the terms “sex differentiation,” “development of primary sexual characteristics,” and “reproductive developmental process.” Similarly, Wu and Zhang reported that reproduction-associated processes (*e*.*g*., “sperm motility,” “spermatid development,” and “gamete generation”) had higher levels of PD [1]. The criteria for HD genes in the present study were more robust than those for genes with higher levels of PD used by Wu and Zhang. Nevertheless, Table 3 reports several terms also identified by Wu and Zhang [1]. For example, terms related to the nervous system (“dendrite” and “neurological system process”), development (“anatomical structure morphogenesis,” “regulation of developmental process,” “system development,” and “cell development”), stress response, homeostasis, growth (“regulation of multicellular organism growth”), secretion (“secretion”), and metabolism had high levels of PD.

The term “behavioral response to nicotine” had the highest fold enrichment of 96.16 (Table 3). This was likely due to the large number of nicotine-related SNPs in our dataset. Specifically, 97 of 654 SNPs were associated with nicotine according to the annotation provided by PharmGKB. The term “adult behavior,” which had a high fold enrichment (15.65) and low *p*-value (0.0075), is an ancestor term of “behavioral response to nicotine.”

Two terms were associated with differentiation in Table 3. “Regulation of cell differentiation” and “cell differentiation” showed fold enrichments of 4.56 and 2.47 and q-values of 0.0157 and 0.0214, respectively.

### 5. GO analysis and pathway analysis: comparison to DR genes

We performed GO analysis and pathway analysis in order to biologically interpret the relationship between the HD gene group and DR genes. The results are summarized as q-values using Benjamini-Hochberg’s method [41] and fold enrichments of the GO terms and pathways. A Benjamini-Hochberg’s q-value less than 0.05 indicates that the HD group contained significantly more genes in the term as compared to randomly selected DR genes.

Table 5 shows the results from the GO analysis and pathway analysis comparing the HD gene group and DR genes. The resulting terms included those related to reproduction that were included in Table 3: “sex differentiation,” “development of primary sexual characteristics,” “reproductive structure development,” “gonad development,” and “development of primary sexual characteristics.”

**Table 5:** Q-values and fold enrichments of hypergeometric test between HD group and drug-related (DR) genes.

Terms	HD vs. others		DR vs. others		HD vs DR	
	FE	BH’s q	FE	BH’s q	FE	BH’s q
BP: Sex Differentiation	8.92	0.0210	2.52	0.2015	3.54	0.0158
BP: Development Of Primary Sexual Characteristics	8.83	0.0468	2.57	0.2558	3.44	0.0283
BP: Reproductive structure development	8.90	0.0504	2.58	0.2531	3.44	0.0283
BP: Gonad development	9.74	0.1139	2.81	0.3622	3.47	0.0527
BP: Development Of Primary Sexual Characteristics	8.83	0.0468	2.57	0.2558	3.47	0.0527
KEGG: Wnt signaling pathway	3.45	0.6823	1.32	0.8715	2.62	0.0556

FE: Fold enrichment

BH’s q: Benjamini-Hochberg’s q-value

Since Wu and Zhang [1] did not conduct a pathway analysis, the “Wnt signaling pathway” was not directly identified. However, their GO analysis identified the term “Wnt receptor signaling pathway through beta-catenin” as statistically significant in the HD gene group [1]. The Wnt signaling pathway is important in pharmacogenetics, because it is strongly associated with cancer [42,43]. Further studies are warranted to identify drugs that inhibit the Wnt signaling pathway, because inhibition of aberrant Wnt signaling in cancer cell lines inhibits their growth [44].

## Discussion

PD is important for understanding differences in drug responses among populations. However, PD often refers to the distance between two different subpopulations; therefore, several studies have investigated approaches for averaging the PD of each SNP. For instance, the impact of SNP ascertainment on estimating the distance between subpopulations has already been reported [45]. In contrast, the present study identified population-specific pharmacogenomics variants. We did not focus on identifying average distances using all SNPs; rather, we used each SNP to identify population-specific pharmacogenomics variants. As a result, our results described the impact of sample ascertainment on different measures of PD for each SNP. In addition, the present study investigated PD of genes in the PharmGKB database, while several previous studies have focused on genes related to individual drugs [5]. This approach enabled us to more systematically study PD of DR genes by considering all reported DR genes from PharmGKB.

In our comparison study, F_st_ showed high specificity and sensitivity robust to the different sample sizes of HapMap release 27. After calculating F_st_ from the allele frequency data of each SNP, we defined HD and LD gene groups. Then, we performed GO analysis and pathway analysis to describe the biological characteristics of the HD gene group. We compared the HD gene group to two different backgrounds: all genes in DAVID and DR genes in the PharmGKB database.

The GO and pathway analyses identified two terms related to cell communication (“cell-cell signaling” and “cell communication”), which had the lowest *p*-values (0.0006 and 0.0002, respectively). In addition, the term “drug binding,” which was related to cell communication, was also considered to be meaningful due to its high fold enrichment (16.51) despite its moderate *p*-value (0.0142). Thus, these results suggest that the HD gene group from PharmGKB is highly associated with cell communication. Since drug binding is associated with the cell membrane, similar to processes related to both cell-cell signaling and cell communication, the simultaneous identification of these GO terms is convincing. In addition, this finding suggests that the cellular location of gene products affects PD. It is possible that, the outer surface of the cell membrane is initially affected by mutagens, because it is closest to the extracellular environment.

In addition, we examined genes containing SNPs with high F_st_ values (above 0.5) among cell-communication-related terms, such as *STX4*, *PPARD*, *DCK*, *GRIK4*, and *DRD3*. Specifically, SNPs rs10871454, rs6922548, rs3775289, rs1954787, and rs167771 had F_st_ values of 0.682, 0.620, 0.573, 0.531, and 0.510, respectively. Further biological studies of these genes will help elucidate their roles in pharmacogenetics.

Unlike other GO analyses, we employed DR genes from PharmGKB and performed an additional analysis by using them as a background for the GO analysis. This strategy of changing the background of the GO analysis from all genes to DR genes represents a novel method. Therefore, our approach has the advantage of providing distinct information about genes of interest by altering the background of analysis.

There are several similarities and differences between the PD study of Wu and Zhang [1] and the present study. Both studies determined PD using F_st_ and investigated characteristics of genes with a high level of differentiation by GO analysis. Thus, the studies identified several similar terms such as those related to replication, development, and metabolism. In addition to the HD gene group, our study performed GO analysis of the LD gene group and compared the results, which identified distinct characteristics of each group. We also conducted GO and pathway analyses comparing the HD gene group to DR genes and identified two meaningful terms, “drug binding” and “Wnt signaling pathway,” which were not identified by Wu and Zhang.

In conclusion, the present study describes an approach for assessing PD associated with multiple drugs using a database. Therefore, the integrated approach may identify valid genetic features different from the background gene list. We validated results from other systematic analyses. Moreover, our approach allows the possibility of improving the results. DR genes that are unknown or newly reported were not included in the present study. Thus, our approach may be limited in its ability to interpret the population-specific difference in drug response or efficacy caused by genetic divergence. However, this method remains convincing, because our statistical analyses revealed high specificity and sensitivity robust to sample size. Furthermore, we obtained significant differences from other DR genes in the PharmGKB database, and our approach thus represents a systematic method for identifying valid population-specific pharmacogenomics variants.
